# *Hox* gene expression in postmetamorphic juveniles of the brachiopod *Terebratalia transversa*

**DOI:** 10.1186/s13227-018-0114-1

**Published:** 2019-01-08

**Authors:** Ludwik Gąsiorowski, Andreas Hejnol

**Affiliations:** Sars International Centre for Marine Molecular Biology, University of Bergen, Bergen, Norway

**Keywords:** Metamorphosis, *Hox* gene collinearity, Indirect development, Morphology, Spiralia, Lophophorata, Biphasic life cycle

## Abstract

**Background:**

*Hox* genes encode a family of homeodomain containing transcription factors that are clustered together on chromosomes of many Bilateria. Some bilaterian lineages express these genes during embryogenesis in spatial and/or temporal order according to their arrangement in the cluster, a phenomenon referred to as collinearity. Expression of *Hox* genes is well studied during embryonic and larval development of numerous species; however, relatively few studies focus on the comparison of pre- and postmetamorphic expression of *Hox* genes in animals with biphasic life cycle. Recently, the expression of *Hox* genes was described for embryos and larvae of *Terebratalia transversa*, a rhynchonelliformean brachiopod, which possesses distinct metamorphosis from planktonic larvae to sessile juveniles. During premetamorphic development, *T. transversa* does not exhibit spatial collinearity and several of its *Hox* genes are recruited for the morphogenesis of novel structures. In our study, we determined the expression of *Hox* genes in postmetamorphic juveniles of *T. transversa* in order to examine metamorphosis-related changes of expression patterns and to test whether *Hox* genes are expressed in the spatially collinear way in the postmetamorphic juveniles.

**Results:**

*Hox* genes are expressed in a spatially non-collinear manner in juveniles, generally showing similar patterns as ones observed in competent larvae: genes *labial* and *post1* are expressed in chaetae-related structures, *sex combs reduced* in the shell-forming epithelium, whereas *lox5* and *lox4* in dorso-posterior epidermis. After metamorphosis, expression of genes *proboscipedia*, *hox3*, *deformed* and *antennapedia* becomes restricted to, respectively, shell musculature, prospective hinge rudiments and pedicle musculature and epidermis.

**Conclusions:**

All developmental stages of *T. transversa*, including postmetamorphic juveniles, exhibit a spatial non-collinear *Hox* genes expression with only minor changes observed between pre- and postmetamorphic stages. Our results are concordant with morphological observation that metamorphosis in rhynchonelliformean brachiopods, despite being rapid, is rather gradual. The most drastic changes in *Hox* gene expression patterns observed during metamorphosis could be explained by the inversion of the mantle lobe, which relocates some of the more posterior larval structures into the anterior edge of the juveniles. Co-option of *Hox* genes for the morphogenesis of novel structures is even more pronounced in postmetamorphic brachiopods when compared to larvae.

**Electronic supplementary material:**

The online version of this article (10.1186/s13227-018-0114-1) contains supplementary material, which is available to authorized users.

## Background

*Hox* genes encode a family of conserved homeodomain transcription factors from the ANTP class, which by binding to regulatory DNA sequences can activate or suppress transcription of downstream genes (e.g., [1, 2]). *Hox* genes are present in genomes of almost all investigated animals (with exception of Porifera, Ctenophora and Placozoa [3–7]) and are hypothesized to represent a synapomorphy of the clade consisting of Cnidaria and Bilateria [4, 8–10]. In most of bilaterians, *Hox* genes are expressed during embryogenesis, being involved in antero-posterior (A-P) patterning of either the whole embryo or at least some of its developing organ systems (e.g., [1, 2, 11]). Interestingly, in the genomes of some animals, the *Hox* genes are clustered along the chromosomes in the same order as they are expressed along A-P axis, a phenomenon referred to as spatial collinearity [2, 11–13]. The clustering of *Hox* genes in the genome is hypothesized as a plesiomorphic feature of Bilateria (e.g., [13]), which, however, went through extensive remodeling in some evolutionary lineages (e.g., [12, 14–22]. Yet, spatial collinearity can be preserved despite a disorganization or split of the ancestral *Hox* cluster (e.g., [14]), the situation for which the term trans-collinearity was coined by Duboule [12].

Initially the role of *Hox* genes has been studied in the developing embryo of *Drosophila melanogaster* [23], later supplemented by the data from other insects, vertebrates and nematodes [24–26]. Recent advance of molecular and bioinformatic techniques allowed the investigation of *Hox* gene expression in the embryos and larvae of several non-model species, including, e.g., xenacoelomorphs [16, 27, 28], hemichordates [29], onychophorans [30], tardigrades [31], rotifers [32], annelids [33–35], mollusks [36–40], nemerteans [41] and brachiopods [19], essentially increasing knowledge on the diversity of *Hox* gene-based patterning systems in Bilateria.

Many animals are characterized by an indirect life cycle in which embryos develop through a larval stage and subsequent metamorphosis, during which the larval body is reshaped into the adult one (e.g., [42, 43]). As larvae and adults can significantly differ in their morphology, the transition process might be quite dramatic and hence attracted attention of many researchers as one of the pivotal moments of the animal development [44–46]. Although the process of metamorphosis has puzzled numerous developmental biologists, there are relatively few studies regarding shifts of *Hox* gene expression accompanying it [15, 47–52]. In some animals, both larvae and adults show canonical spatial collinearity, which often correlates with the gradual type of metamorphosis. This can be exemplified by investigated annelid species, in which both life stages exhibit spatial collinearity of most of the *Hox* genes, yet there are shifts in the combinations of genes defining particular body regions before and after metamorphosis [47, 48]. On the other hand, in other animals (especially those with the more pronounced metamorphosis) only one of the developmental stages exhibits canonical spatial collinearity of *Hox* genes expression, whereas the remaining stage shows either a non-collinear expression or does not express *Hox* genes at all. For instance, in the tunicate *Ciona intestinalis Hox* genes exhibit spatially collinear expression in the nervous system of larvae, whereas in juveniles only the three posterior genes are expressed in the intestine [15]. Conversely in pilidiophoran nemertean *Micrura alaskensis* and in indirectly developing enteropneust *Schiozcardium californicum* the specialized larvae develop without expressing any of the *Hox* genes, which, in turn, are expressed in the canonical collinear way only in the rudiments of juvenile worms developing either inside larval body (pilidiophorans) or as the posterior extension of late larva (enteropneusts) [49, 50]. A somehow similar situation is found in the indirectly developing sea urchin *Strongylocentrotus purpuratus*, in which only two *Hox* genes (*hox7* and *hox 11/13b*) take part in the larva formation, whereas the rudiments of adult animal, developing inside the larval body, show collinear expression of five *Hox* genes (*hox7*, *hox8*, *hox9/10*, *hox11/13a* and *hox11/13b*) in the extra-axial mesoderm [51, 53–55]. Yet another type of the metamorphosis-related *Hox* genes expression shifts is found in scaphopod *Antalis entalis* in which only the mid-trochophore stage exhibits staggered *Hox* genes expression, whereas both competent larvae and postmetamorphic juveniles lack spatial collinearity [52]. Some of the scaphopod *Hox* genes partially retain their expression profiles throughout metamorphosis (*hox2*, *hox5*, *lox5*), whereas other substantially changes their expression domains (*hox3*, *lox4*, *post1*, *post2*) or are expressed only before (*hox1*) or after (*hox4*) metamorphosis [52]. It is therefore evident that the metamorphosis-related shifts in *Hox* gene expression and function vary a lot from one animal clade to another, as a result of diverse evolutionary and developmental processes, which shape the ontogeny of each particular group [56].

**Fig. 1:**
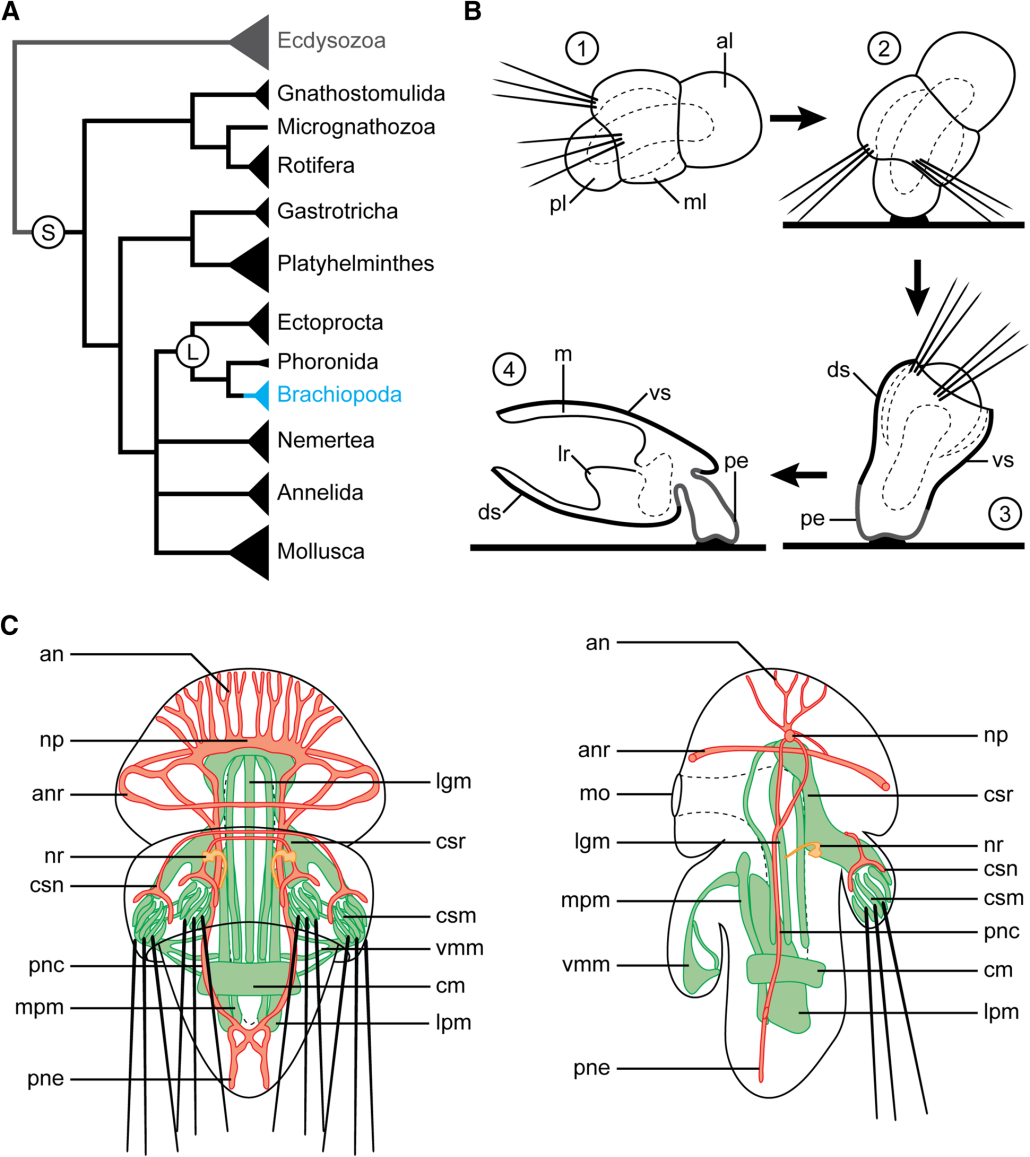
Phylogenetic position of Brachiopoda (**A**, based on Laumer et al. [58]), metamorphosis of *Terebratalia transversa* (**B**, based on Freeman [68]) and detailed morphology of competent larva (**C**, based on Santagata [70]). S stands for Spiralia, L for Lophophorata. *1* Competent planktonic larva, anterior to the right; *2* Larva settles on the substrate; *3* Inversion of the mantle lobe in the settled larva; *4* Juvenile; note that over the course of metamorphosis the internal surface of the larval mantle lobe becomes external, shell-covered surface of juvenile animal, external surface of the mantle lobe becomes inner surface of the mantle, whereas anterior lobe contributes to the lophophore rudiment developing inside mantle cavity. Musculature in C is depicted in green, nervous system in red and excretory organs in orange. *al* larval anterior lobe, *an* anterior nerves, *anr* anterior nerve ring, *cm* circular muscle, *crs* chaetal sac retractor muscle, *csm* chaetal sac musculature, *csn* chaetal sac nerve, *ds* dorsal shell, *lgm* longitudinal gut-related muscles, *lpm* lateral pedicle muscle, *lr* lophophore rudiment, *m* mantle, *ml* larval mantle lobe, *mo* mouth, *mpm* medial pedicle muscle, *np* neuropil, *nr* nephridium rudiment, *pcn* paraxial nerve cord, *pe* pedicle, *pl* larval pedicle lobe, *pne* pedicle nerve, *vmm* ventral mantle lobe lateral muscle, *vs* ventral shell

One of the animal groups with a distinct metamorphosis event are rhynchonelliformean brachiopods, represented by *T. transversa* for which Schiemann et al. recently described *Hox* genes expression in embryos and larvae [19]. Brachiopods, along with phoronids and possibly ectoprocts, constitute the clade Lophophorata (Fig. 1A, [57, 58]), which, together with, for example, annelids, mollusks, flatworms, nemerteans and rotifers, belongs to a large clade of protostome animals called Spiralia (Fig. 1A, [58–61]). Extant brachiopods are traditionally divided into three groups: Rhynchonelliformea, Craniiformea and Linguliformea, the two latter forming sister clades [18, 58, 61, 62], historically united into group Inarticulata. As all brachiopods, adults of *T. transversa* are filter feeding animals with external anatomy superficially similar to bivalves—most of the body, including lophophore, a filtering organ, is enclosed in the two-valved shell, which covers the dorsal and ventral surfaces of the body. The clade Rhynchonelliformea is further characterized by the set of morphological features, including posterior soft-tissued pedicle (by which animal attaches to the substrate), blind gut devoid of anus and articulated valve-hinge [63]. Additionally, rhynchonelliformean larvae (Fig. 1B1, C) differ from those found in other brachiopods by possessing three distinct body regions—anterior lobe, mantle lobe (bearing four chaetal sacs) and the most posterior pedicle lobe [63–67]. The rhynchonelliformean larva settles by adhering to the substrate with the posterior tip of the pedicle lobe (Fig. 1B2) and undergoes a specific metamorphosis, which in case of *T. transversa* is relatively rapid (few hours to 1 day [64]) and involves inversion of the mantle lobe (Fig. 1B2–3, [64, 68]). The latter results in profound relocation of some larval tissues—in competent larvae the mantle lobe partially covers the pedicle one and its chaetae projects posteriorly, after metamorphosis mantle lobe with chaetae projects anteriorly, its former interior surface becomes exposed and produce protegulum (the first rudiment of the shell), whereas its former exterior surface constitutes walls of the mantle cavity of the juvenile (Fig. 1B2–4) [64, 68, 69]. Therefore, the rapid transition from larvae to the juvenile involves profound reshaping of the entire body, which poses a question to which extent are those two stages continuous [70].

**Fig. 2:**
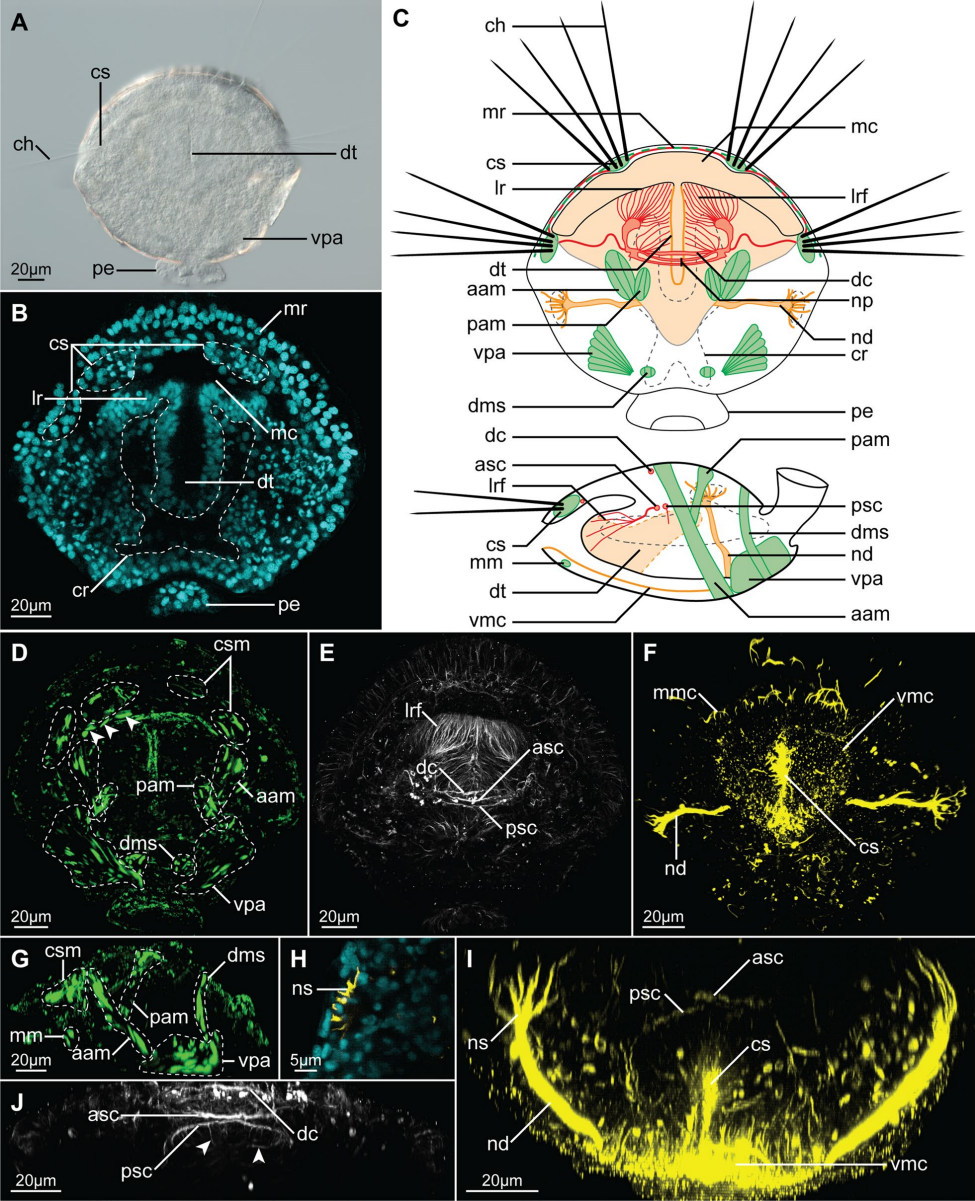
Morphology of the juvenile of *Terebratalia transversa* (2 days after metamorphosis), visualized with light microscopy (**A**) and CLSM (**B**, **D**–**I**). **A** Micrograph of the entire animal. **B** Frontal section through the median part of animal, cell nuclei visualized with DAPI staining. **C** Schematic drawing of the anatomy of juvenile in dorso-ventral (top) and lateral (bottom) views, musculature in green, tyrosinated-tubulin immunoreactive nervous system in red, acetylated-tubulin immunoreactive structures in orange (mantle margin ciliation not shown for clarity). **D**, **G** Musculature visualized with F-actin phalloidin staining, arrowheads in **D** point to the ventral mantle margin muscles. **E**, **J** Tyrosinated-tubulin immunoreactivity. **F**, **I** acetylated-tubulin immunoreactivity. **H** Transverse section through the nephrostome, cell nuclei visualized with DAPI in cyan, acetylated-tubulin immunoreactivity in yellow. **A**–**F** Dorso-ventral view, anterior to the top. **G** Lateral view, anterior to the left, dorsal to the top. **I** Virtual transverse section through the middle part of animal, dorsal to the top. **J** Ventral, magnified view of the commissural region from **E**. *aam* anterior shell adductor muscle, *asc* anterior supraesophageal commissure, *ch* chaeta, *cr* coelom rudiment, *cs* chaetal sac, *csm* chaetal sac musculature, *dc* dorsal commissure, *dms* shell diductor muscle, *dt* digestive tract, *lr* lophophore rudiment, *lrf* fibers in the lophophore rudiment, *mc* mantle cavity, *mm* mantle margin musculature, *mmc* mantle margin ciliation, *mr* mantle margin, *nd* nephroduct, *np* neuropil, *ns* nephrostome, *pam* posterior shell adductor muscle, *pe* pedicle, *psc* posterior supraesophageal commissure, *vmc* ventral mantle cavity ciliation, *vpa* ventral pedicle adjustor muscle

**Fig. 3:**
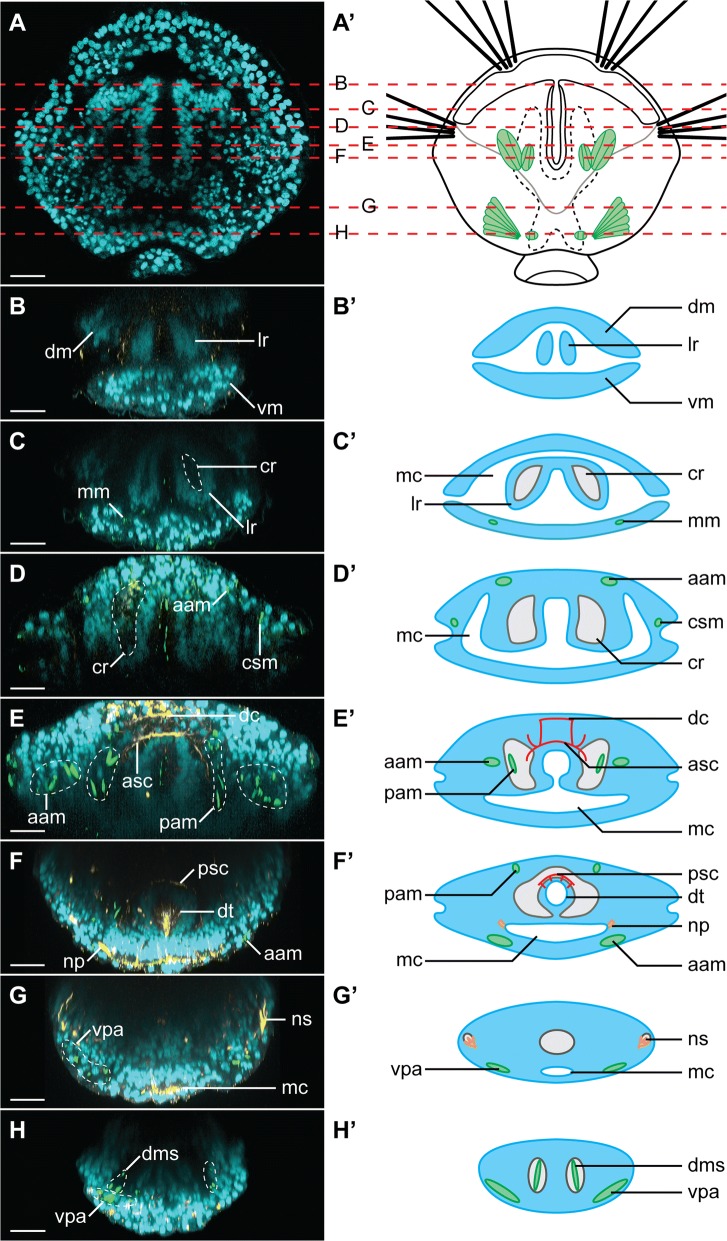
Transverse sections through *Terebratalia transversa* juvenile (2 days after metamorphosis), showing detailed morphology of the animal. Each panel consists of CLSM image (left) and schematic representation (right). Cell nuclei visualized with DAPI in cyan, acetylated-tubulin immunoreactivity (**F** and **G**) and tyrosinated-tubulin immunoreactivity (**E**) in yellow, F-actin phalloidin staining in green. **A** Juvenile in dorso-ventral view, anterior to the top, dashed red lines indicates sections shown on the subsequent panels. **B**–**H** Virtual transverse sections through the juvenile, as indicated on **A**, dorsal to the top on all panels, on the right panels outline of the body is depicted in light blue, musculature in green, body cavities in gray, nervous system in red and excretory organs in orange. Scale bars on all images represent 20 μm. *aam* anterior shell adductor muscle, *asc* anterior supraesophageal commissure, *cr* coelom rudiment, *csm* chaetal sac muscle, *dc* dorsal commissure, *dm* dorsal mantle, *dms* shell diductor muscle, *dt* digestive tract, *lr* lophophore rudiment, *mc* mantle cavity, *mm* mantle margin muscle, *np* nephropore, *ns* nephrostome, *pam* posterior shell adductor muscle, *psc* posterior supraesophageal commissure, *vm* ventral mantle, *vpa* ventral pedicle adjustor muscle

**Fig. 4:**
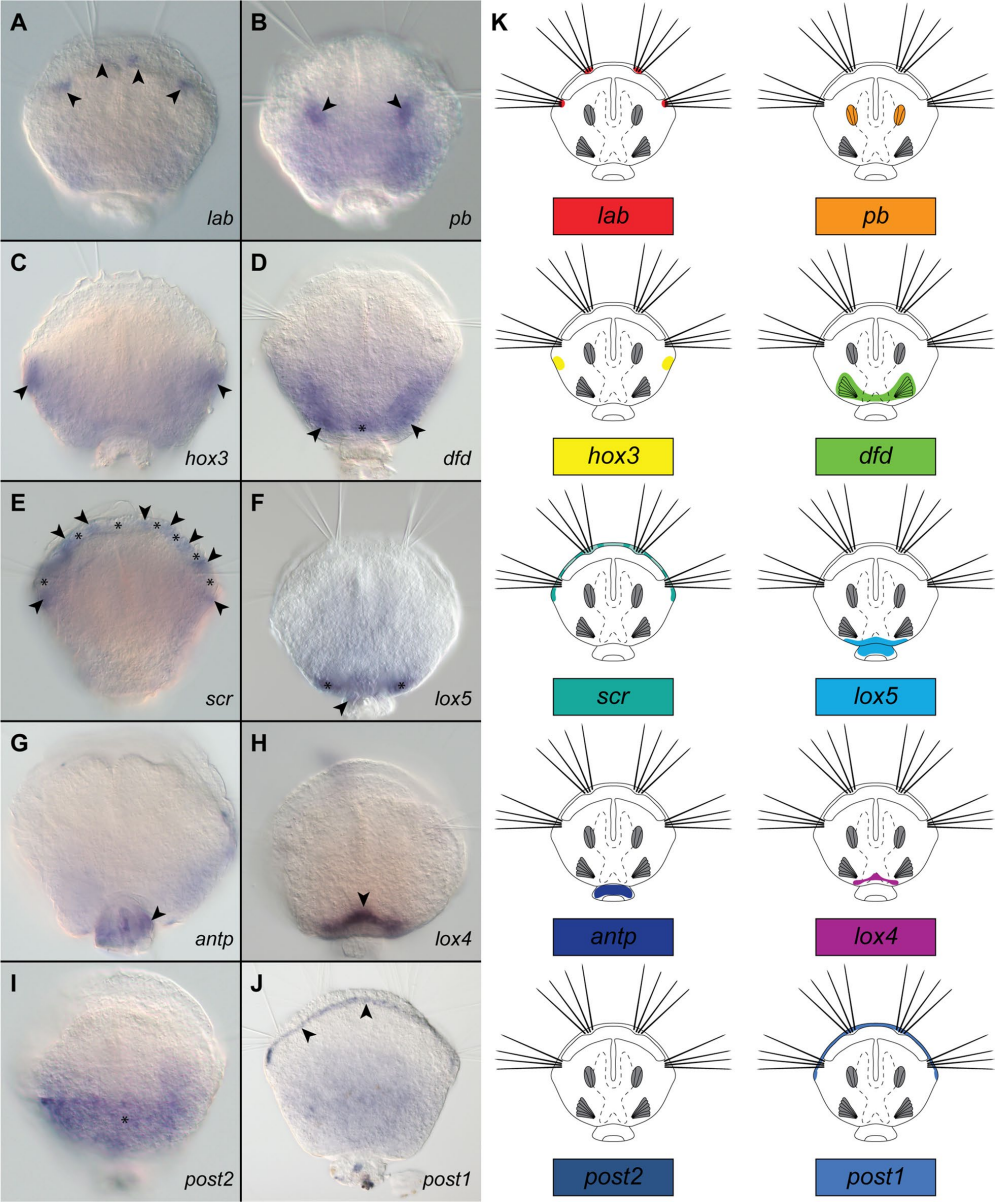
Whole-mount colorimetric in situ hybridization of the *Hox* genes (**A**–**J**) and schematic representation of expression patterns (**K**) in *Terebratalia transversa* postmetamorphic juveniles (2 days after metamorphosis). All micrographs and drawings in dorso-ventral view, anterior to the top. For each plate (**A**–**J**) name of the hybridized gene is provided in the lower right corner. Particular structures in which each of the genes is expressed are indicated with arrowheads, asterisks and double arrowheads (see text for detailed explanation). Note that signal on I (asterisk) represents unspecific background (see text for details)

Schiemann et al. investigated genomic order of *Hox* genes of *T. transversa* and *Hox* genes expression in embryos and larvae of *T. transversa* and craniiformean *Novocrania anomala* [19]. *T. transversa* has a split *Hox* cluster comprising of 10 *Hox* genes in three independent parts. One scaffold contains two anterior *Hox* genes, *labial* (*lab*) and *proboscipedia* (*pb*). A separate scaffold contains the longest section of the *Hox* complex, containing genes *hox3*, *deformed* (*dfd*), *sex combs reduced* (*scr*), *lox5*, *antennapedia* (*antp*), *lox4* and *post2*, whereas the most posterior gene *post1* is located in the third independent scaffold [19]. A disorganization of the *Hox* cluster has also been reported for linguliformean brachiopod, *Lingula anatina*, in which although all *Hox* genes are in the single cluster *post1*, *post2*, *lox4* and *antp* have been translocated upstream to the *lab* [18]. In embryos and larvae of *T. transversa*, but also of craniiformean *N. anomala*, detected expression pattern of *Hox* genes does not show the canonical spatial collinearity [19]. However, as stated before, in some indirectly developing animals, larvae and juveniles can show collinear expression of *Hox* genes in patterning of one of the life stages, while the other develops without evident *Hox* expression collinearity.

Therefore, in this study, we supplemented findings of Schiemann et al. [19] by examination of the postmetamorphic *Hox* gene expression in *T. transversa* juveniles 2 days after metamorphosis. The main questions, which we were aiming to answer, were: (1) If and how is *Hox* genes expression pattern shifted during metamorphosis in rhynchonelliformean brachiopods? (2) Is there any staggered *Hox* genes expression along the A-P axis emerging after metamorphosis as a result of displacement of larval Anlagen and their development into definite adult structures?

## Results

### Description of *T. transversa* juvenile morphology

Existing knowledge of the detailed morphology of the juvenile *T. transversa* is based mostly on the confocal laser scanning microscopy (CLSM) investigation of musculature [70, 71], as well as transmission electron microscope (TEM) sections [64, 69, 72] and scanning electron microscopy (SEM) [69, 72, 73] of different developmental stages, including juveniles 1 day after metamorphosis [69, 70, 73], 4–5 days after metamorphosis [69, 71–73] and older than 1 week after metamorphosis [69, 70, 72, 73]. Therefore, to facilitate interpretation of our gene expression results [74], we examined morphology of the juveniles 2 days after metamorphosis utilizing light microscopy (LM) and CLSM combined with DAPI, phalloidin and immunohistochemical stainings (with primary antibodies against tyrosinated and acetylated tubulin).

Two days after metamorphosis, the juveniles of *T. transversa* already resemble the adult animal in their general shape (Fig. 2A–C). The body is clearly divided into main part covered by the two-valved juvenile shell and a posterior pedicle (*pe*, Fig. 2A–C), by which the juvenile is attached to the substrate.

Anteriorly, the shell is lined with the mantle margin (*mr*, Fig. 2B, C), where the tissues responsible for the secretion of the prospective adult shell are localized [69]. Phalloidin staining revealed the presence of the developing mantle margin muscles (arrowheads Fig. 2D; *mm*, Figs. 2G, 3C, C’), which have been already described for the older juveniles [70, 71]. Additionally, four chaetal sacs (*cs*, Fig. 2A–C), associated with the degenerating larval musculature (csm, Figs. 2D, G; 3D, D’) [70], are embedded in the dorsal mantle margin, one pair dorso-medially and another in the more lateral position, which, respectively, protrude numerous chaetae (*ch*, Fig. 2A, C) anteriorly and laterally.

Optical sections through the animal show the narrow mantle cavity (*mc*, Figs. 2B, C, 3C’–G’), which expands ventro-medially to about two-thirds of the length of the animal body and is lined with the ciliated cells (*vmc*, Fig. 2C, F, I). The remnant of the larval anterior lobe, from which the prospective lophophore will develop [66, 75], is situated inside the mantle cavity (*lr*, Figs. 2B, C, 3B, B’, C, C’). Posteriorly the lobe is connected with the dorsal mantle, and ventrally it faces the extension of the mantle cavity (Fig. 3D, D’, E, E’). Medially the lobe is divided by the ciliated slit (*cs*, Fig. 2F, I), which anteriorly communicates with the mantle cavity through the ventral infold, a stomodeum (Fig. 3 C–E, C’–E’) and posteriorly continues as the tubular rudiment of the digestive tract (*dt*, Figs. 2A–C, 3F, F’).

At this stage, the lophophore rudiment is poorly developed and consists of two, scarcely ciliated lobes without tentacles (*lr*, Figs. 2B, C, 3B, B’, C, C’). The lobes are penetrated by numerous, fine tyrosinated-tubulin immunoreactive (tTIR) fibers (*lrf*, Fig. 2C, E), which communicate with the nervous system and probably represent the developing innervation of the prospective lophophore.

The most prominent structure of the nervous system is the brain neuropile (*np*. Fig. 2C), which consists of two tTIR and acetylated-tubulin immunoreactive (aTIR) commissures (anterior and posterior supraesophageal commissures, respectively, *asc* and *psc*, Figs. 2C, E, I, J, 3E, E’, F, F’), positioned dorsally to the ciliated slit. Some tTIR and aTIR fine fibers extend laterally from those commissures to the lophophore rudiments (lrf, Fig. 2C, E) and mantle tissues (including mantle margin and chaetal sacs, Fig. 2C). Few tTIR fine neurites extend from the posterior supraesophageal commissure to the intestinal tissue (*arrowheads*, Figs. 2J, 3F’). Additional dorsal tTIR commissure (*dc*, Figs. 2C, E, J, 3E, E’) connects with the anterior supraesophageal commissure. The similar arrangement of the nervous system in the early juveniles of *T. transversa* has been reported based on immunostaining against serotonin [28].

Two prominent tTIR and aTIR longitudinal structures are present in the ventro-lateral part of the animal (*nd*, Fig. 2C, F, I), extending along the ventral surface from the mid-posterior region to the ventro-posterior part of the mantle cavity. Dorsally those structures have numerous finger-like projections (*ns*, Figs. 2H, I, 3G, G’), which contact nuclei-free regions (as revealed by DAPI staining, Fig. 2H). We suggest that those structures represent metanephridia (composed of nephrostome and nephridial duct) of the juveniles, which connect the developing coelom with the mantle cavity. Their form and position are similar to what has been described for the metanephridia of relatively closely related *Terebratulina retusa* [76]. Although metanephridia in brachiopods are considered to be responsible only for release of gametes and not for excretion [76], they are present (albeit initially as non-functional rudiments) already in the early juveniles of *N. anomala* [77]. It is possible that aTIR structures described by Santagata [70] as larval protonephridia in *T. transversa* (*nr*, Fig. 1C) actually represent rudiments of the metanephridial ducts or nephrostomes which acquire their final form during or soon after metamorphosis.

The DAPI staining revealed an empty cavity inside the body of the juvenile with two pairs of anterior and posterior branches (cr, Figs. 2B, C, 3), which most probably represents the developing coelom in which some of the forming muscles are freely positioned (Fig. 3E, E’, H, H’) [78]. Its two anterior branches extend along digestive tract and penetrate the lophophore rudiment (Figs. 2B, 3C–F, C’–F’). A similar arrangement of the coelom in the lophophore rudiment of postmetamorphic juveniles has been described for relatively closely related rhynchonelliformean *Calloria inconspicua* [79].

In addition to the already mentioned musculature related to the mantle margin, we identified rudiments of all the muscle groups (pedicle adjustors, shell diductors as well as anterior and posterior shell adductors, respectively, vpa, dms, aam, pam, Figs. 2D, G, 3D–H, D’–H’) described for the older juveniles of *T. transversa* [71] with the only exception of the lophophore-related tentacle muscles (which correlates with lack of the lophophore tentacles 2 days after metamorphosis). In different specimens, the particular groups of muscles were developed to different degree corroborating the observation of the extensive and rapid remodeling of muscular tissue in the postmetamorphic juveniles [70].

### In situ hybridization of *Hox* genes

**Fig. 5:**
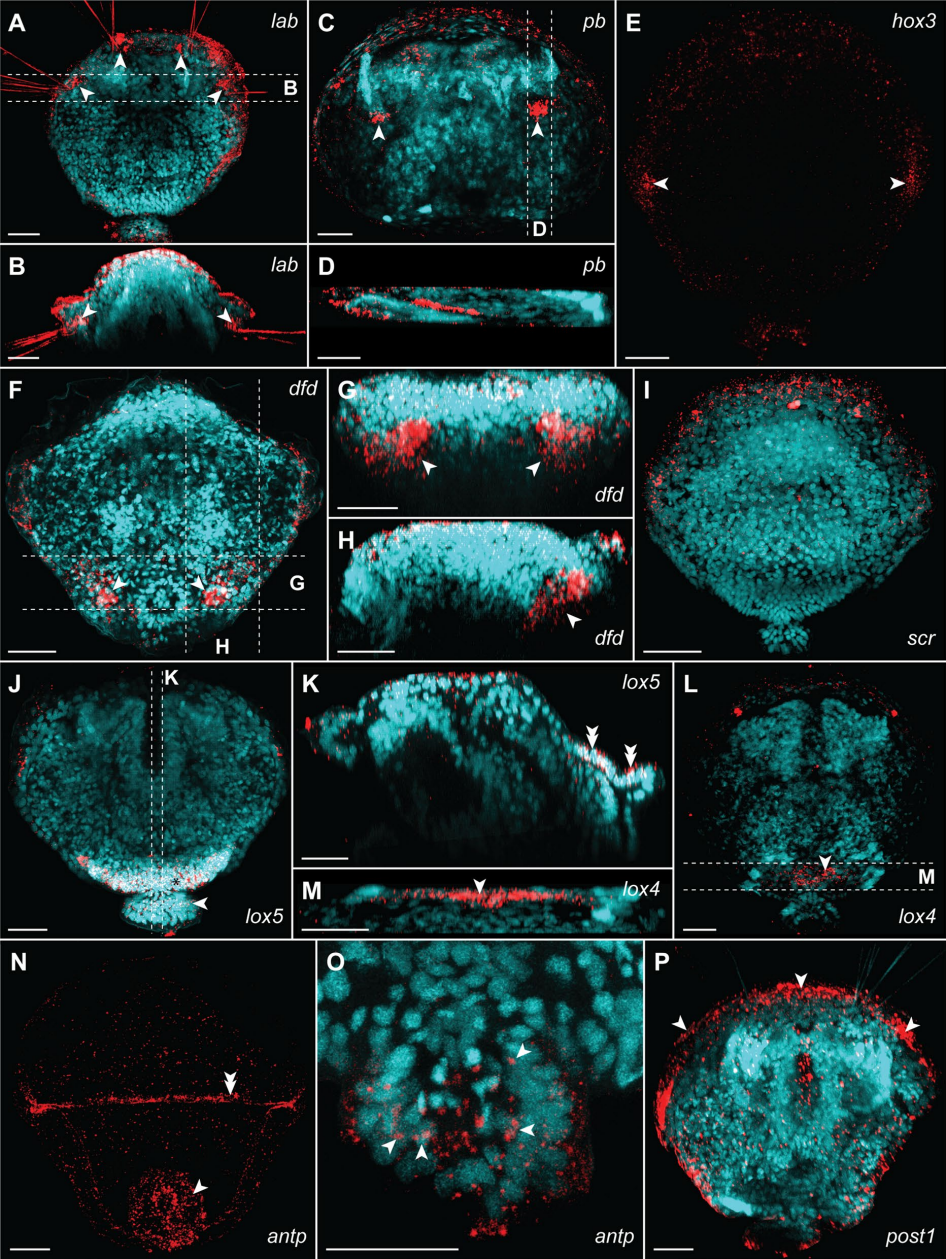
Whole-mount fluorescent in situ hybridization of the *Hox* genes (red) combined with DAPI staining of cell nuclei (cyan, with exception of **E** and **N**) in *Terebratalia transversa* postmetamorphic juveniles (2 days after metamorphosis). For each plate name of the hybridized gene is provided in the right corner. Particular structures in which each of the genes is expressed are indicated with arrowheads, asterisks and double arrowheads (see text for detailed explanation). Expression of *lab* in chaetal sacs in dorso-ventral view (**A**) and virtual cross section (**B**); note autofluorescence of chaetae. Expression of *pb* in anterior shell adductor muscles in dorso-ventral view (**C**) and virtual parasagittal section (**D**). Expression of *hox3* in dorso-ventral view (**E**). Expression of *dfd* in ventral pedicle adjustor muscles in dorso-ventral view (**F**), virtual cross section (**G**) and parasagittal section (**H**). Expression of *scr* in mantle margin (**I**). Expression of *lox5* in dorso-posterior epidermis in dorso-ventral view (**J**) and virtual sagittal section (**K**). Expression of *lox4* in dorso-posterior epidermis in dorso-ventral view (**L**) and virtual cross section (**M**). Expression of *antp* in pedicle tissue in dorso-ventral view (**N**, **O**). Expression of *post2* in mantle margin, dorso-ventral view (**P**). Dashed lines with letters on **A**, **C**, **F**, **J**, **L** and **O** indicate section planes shown on respective plates. Scale bars on all images represent 20 μm. Anterior to the top on **A**, **C**, **E**, **F**, **I**, **J**, **L**, **N** and **R**; dorsal to the top on **B**, **D**, **G**, **H**, **K** and **M**; anterior to the left on **D**, **H** and **K**.

**Fig. 6:**
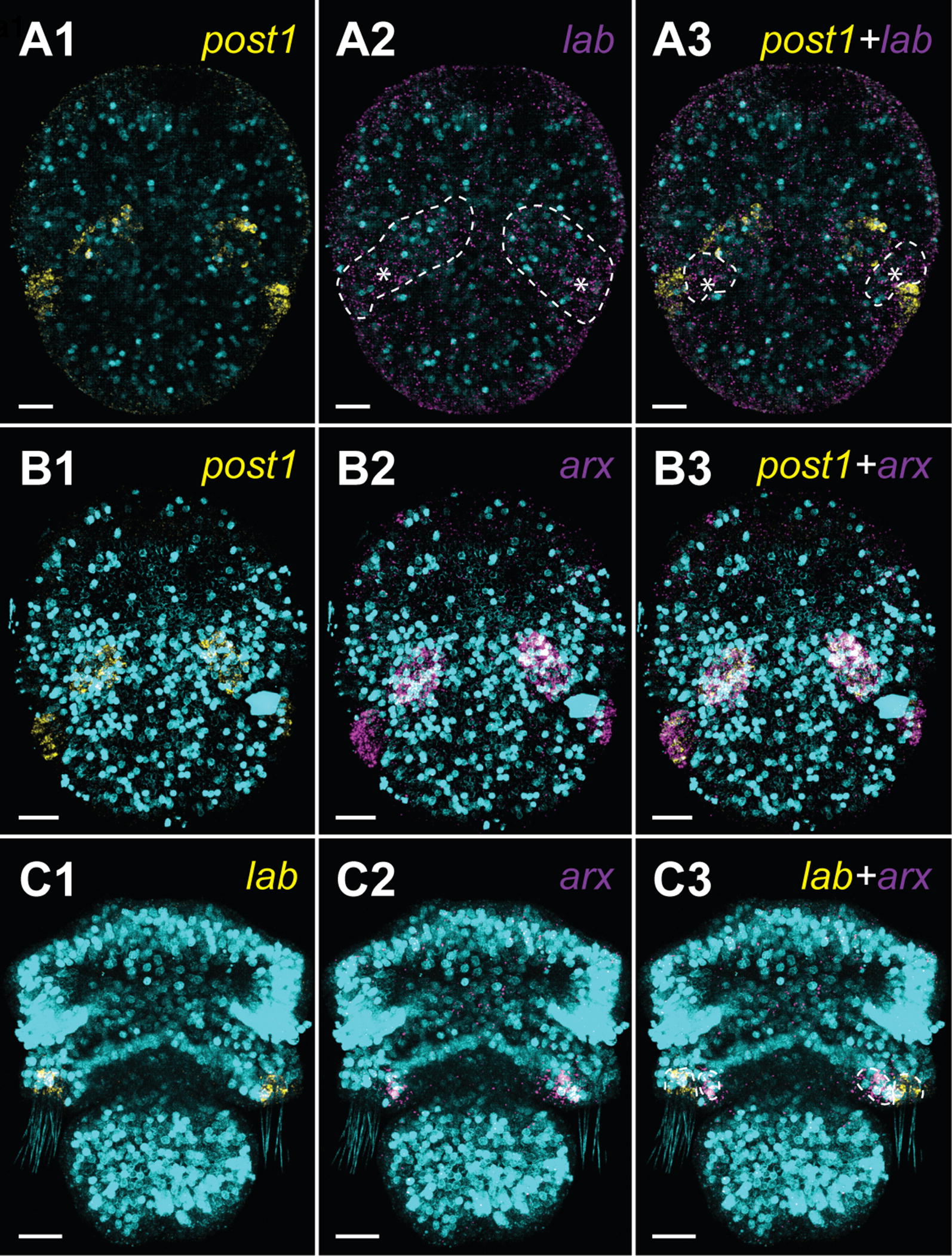
Expression of the chaetae-related genes in the early developmental stages of *Terebratalia transversa* combined with DAPI staining of cell nuclei (cyan). Double fluorescent in situ hybridization of *post1* and *lab* (**A**), *post1* and *arx* (**B**) and *lab* and *arx* (**C**) in the early gastrulae (**A**, **B**) and the early trilobed larva (**C**). Dorso-ventral view and anterior to the top on all panels. Scale bars on all images represent 20 μm

The expression of the *Hox* genes in juvenile *T. transversa* (2 days after metamorphosis) was examined with colorimetric (CISH; Fig. 4) and fluorescent (FISH, Fig. 5) in situ hybridization. *Hox* genes in the juveniles of *T. transversa* are not expressed in a strictly collinear way (Figs. 4, 5, 6).

The most anterior *Hox* gene *lab* is expressed in the two bilaterally paired domains at the mantle margin, which correspond to the larval chaetal sacs (arrowheads, Figs. 4A, 5A, B; also compare with Fig. 2B, D).

The gene *pb* has bilaterally paired strong expression domains (arrowheads, Figs. 4B, 5C), which correspond to the position in which shell adductor muscles are developing (compare Figs. 2D, 4B, 5C). The FISH and CLSM investigation of juveniles further revealed that those two domains extend obliquely from the more anterior point on the dorsal shell to the more posterior point on the ventral shell, in the same orientation as the anterior shell adductors (compare Figs. 2C, G, 5D).

*hox3* is expressed in two paired domains posteriorly to the most lateral projections of the shell (arrowheads, Figs. 4C, 5E), where prospective hinge rudiments will form in the older juveniles [69]. CISH investigation showed additional broad weak staining in the posterior part of the body (Fig. 4C), which was not reproduced with FISH (Fig. 5E) and which might result from unspecific probe binding in the posterior shell as shown by sense probe staining (Additional file 1: Fig. S1B and C).

The gene *dfd* is expressed in the ventro-posterior domain (Figs. 4D, 5F–H) composed of extensive lateral elements (arrowheads, Figs. 4D, 5F–H), which merge posteriorly (connection visible only with CISH, asterisks Fig. 4D). Position of those structures revealed with FISH (Fig. 5F–H) indicates that *dfd* is expressed in the ventral pedicle adjustor muscles (compare Figs. 2C, D, G, 5F–H).

Expression of the gene *scr* is restricted to the mantle margin (Figs. 4E, 5I). Signal from probes against *scr* in CISH seems to be diversified into smaller domains with strong signal interspaced by wider regions of relatively weaker expression (respectively, arrowheads and asterisks, Fig. 4E), indicating an unequal expression of the gene along mantle margin. However, this diversification is not visible in FISH examination (compare Figs. 4E, 5I). The uniform signal from probes against *scr* in FISH might be an effect of the specific staining of the *scr* expressing cells and unspecific binding of the probe at the mantle margin (as in fluorescent stainings against *lab* and *pb*, where unspecific signal is visible along mantle margin; compare Fig. 5I with 5A, C).

The gene *lox5* is expressed in the continuous dorso-posterior domain, which extend from posterior region of the shell-covered body (asterisks, Figs. 4F, 5J) to the pedicle tissues (arrowheads, Figs. 4F, 5J) and its expression is restricted to the dorsal epidermal cells as revealed by FISH (double arrowheads, Fig. 5K).

*antp* has a distinct expression domain only in the epidermis of the pedicle, as revealed by both CISH (arrowhead, Fig. 4G) and FISH (arrowhead, Fig. 5N–P). The signal in the CISH staining developed for the long time and before it became evident the strong staining had appeared in some specimens also in the dorso-posterior part of the shell-covered body. However, the control with sense probe showed that this staining results from unspecific binding of the probe in the dorsal protegulum (larval shell rudiment; asterisks, Additional file 1: Figure S1B) and on the borders between the dorsal protegulum and the remaining parts of the shell (arrowheads, Additional file 1: Figure S1B). The strong dorsal band was also visible in FISH staining (double arrowheads, Fig. 5N), but combined staining with DAPI showed that it is restricted to the surface area and does not penetrate the epidermis (arrowhead, Additional file 1: Fig. S1D, E), supporting our finding that it represents an unspecific probe binding by shell components.

The central-class *Hox* gene *lox4* is expressed only in the small area of epidermal tissues in the dorso-posterior part of the shell-covered body (arrowheads, Figs. 4H, 5L, M), and its expression domain is not extending to the pedicle tissues.

We did not manage to detect expression of *post2* with in situ hybridization, which corresponds to the reported overall low level of *post2* transcription in postmetamorphic juveniles of *T. transversa* [19]. After long developmental time, CISH staining yielded signal in the dorso-posterior part of the shell-covered body (asterisk, Fig. 4I); however, the control with the sense probe showed that this results from unspecific binding of the probe in the larval dorsal protegulum (Additional file 1: Figure S1C). The FISH staining only revealed a signal at the borders of the larval protegulum and the remaining parts of the shell (arrows, Additional file 1: Fig. S1F) and, similarly as in case of *antp*, FISH combined with DAPI staining revealed that this signal is restricted to the surface (shell components) and does not penetrate to the cellular epidermal layer (arrowhead, Additional file 1: Figure S1G). The unspecific binding of some probes by the larval protegulum has been already reported for *T. transversa* larvae [80], and apparently this phenomenon can also pose a problem in investigation of postmetamorphic animals.

Expression of the most posterior *Hox* gene *post1* is detected along mantle margin (arrowheads, Figs. 4J, 5R), showing a relatively equal strength of signal with both CISH and FISH.

### Double fluorescent in situ hybridization of the chaetae-related genes

In addition to the investigation of *Hox* genes in postmetamorphic juveniles, we performed double FISH of genes *lab, post1* and *arx* (Aristaless-related homeobox) at the early developmental stages of *T. transversa* in order to better understand the relation of the expression patterns to the chaetal sac formation. The two former *Hox* genes have been proposed as related to chaetae formation in Brachiopoda [19], whereas *arx* is expressed in the chaetal sac musculature of annelid *Platynereis dumerilli* [81] and in the developing chaetal sacs of *T. transversa* [19]. We did a double fluorescent staining of *post1* and *labial* (Fig. 6A) as well as *post1* and *arx* (Fig. 6B) in the late gastrula stage and *lab* and *arx* in the early trilobed larva (Fig. 6C).

In the late gastrulae, gene *post1* is co-localized with *lab* (Fig. 6A3), which shows extremely weak expression at this developmental stage (asterisks, Fig. 6A2). This is concordant with the CISH results from Schiemann et al. [19]), but our results show that it is also expressed in some *post1*-negative cells in between chaetal sac Anlagen (asterisks, Fig. 6A3). Additionally *post1*-positive cells of the late gastrulae strongly express gene *arx* (Fig. 6B). In the early trilobed larvae *lab* is expressed in the chaetal sac-related cells (Fig. 6C1), whereas *arx* expression is restricted to the subpopulation of the cells of the inner mantle lobe epithelium (Fig. 6C2) and the two genes are not co-expressed by any cells (Fig. 6C3).

## Discussion

### Metamorphosis and *Hox* gene expression in Rhynchonelliformea

**Fig. 7:**
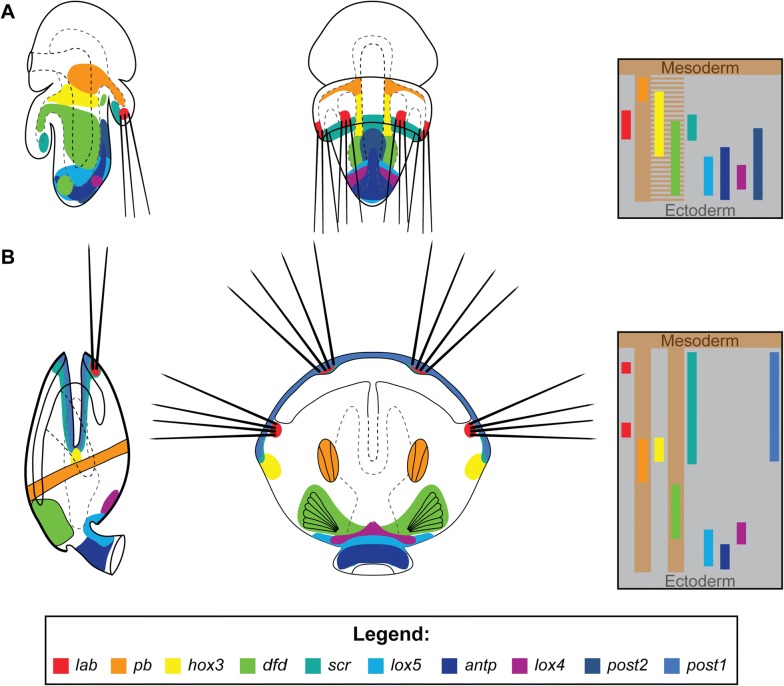
Comparison of the *Hox* genes expression between the late competent larva (**A**, based on Schiemann et al. [19]) and juvenile (**B**) of *Terebratalia transversa*. Animals are shown in the dorso-ventral view (right panels) and in the lateral view with dorsal to the right (left panels). Anterior to the top on all panels. Bars on the right show antero-posterior *Hox* gene expression gradients in ectoderm and mesoderm of each developmental stage

Comparison of the expression of *Hox* genes between late, competent larva and postmetamorphic juvenile of *T. transversa* (Fig. 7) shows that in both stages almost all *Hox* genes (with the exception of *hox3*, *post2* and *post1*) are expressed in the corresponding organs and body regions: *lab* in chaetal sacs, *pb* and *dfd* in mesoderm, *scr* in the shell growth zone, whereas *lox5*, *antp* and *lox4* are expressed in the dorso-posterior ectoderm. Most of the observed differences and shifts in the expression domains can be explained by the inversion of the mantle lobe, which constitutes the most profound process during the whole metamorphosis in Rhynchonelliformea (Fig. 1B, [64]). Another factor, which contributes to the observed changes, is the restriction of the expression of some *Hox* genes from broad, less specific larval domains to the particular structures of the juvenile, which emerge during or after metamorphosis. For example, *pb* is generally expressed in the anterior mesoderm in late larvae but in juveniles its expression becomes restricted only to particular mesodermal structures, i.e., newly formed anterior shell adductors muscles.

The comparison of the *Hox* gene expression between larvae and juveniles allows the identification of the Anlagen of adult structures in the larva. For example, the expression patterns of the *Hox* genes before and after metamorphosis suggest that only the posterior part of the larval pedicle lobe contributes to the pedicle of the adult, whereas the more anterior part becomes the posterior region of the shell-covered body, as it has been proposed by Stricker and Reed [64, 72]. Among six *Hox* genes expressed in the pedicle lobe of the late larvae of *T. transversa*, only *lox5* and *antp* are expressed in the pedicle of the postmetamorphic juvenile (Fig. 7), both of them being expressed in the most posterior part of the larval pedicle lobe [19].

Next to Rhynchonelliformea, two inarticulate clades belong to Brachiopoda: Craniiformea and Linguliformea [82], both possessing a planktonic larvae, which undergoes more or less pronounced metamorphosis [63, 70, 83–87]. In Linguliformea, the metamorphosis itself is extended over time with some of the juvenile traits present already in the planktotrophic larvae [67, 70, 83, 88], and the most advanced larval stages are even commonly considered as representing planktonic juveniles or paralarvae [63, 70, 88]. One can therefore speculate that as larval and adult body plans in Linguliformea are continuous, their patterning by *Hox* genes should be similar as is a case in *T. transversa*. On the other hand, there are two competing hypotheses about nature of the rearrangement of the larval body plan during metamorphosis of craniiformean brachiopods [63, 84, 85, 89, 90]. The main controversy regards whether the *N. anomala* larva, which lacks the distinct pedicle lobe, attaches to the substrate with its dorso-posterior side [84] or with the posterior tip of the posterior lobe [85]. Expression of the *Hox* genes is relatively similar between embryos and larvae of *N. anomala* and corresponding stages of *T. transversa* [19], indicating a conserved nature of *Hox* genes patterning between Craniiformea and Rhynchonelliformea. *lox5* and *antp*, which after metamorphosis are expressed in the pedicle of *T. transversa* juveniles are expressed in the posterior tip of the posterior lobe of *N. anomala* larvae [19] favoring interpretation that posterior tip of *N. anomala* larvae corresponds to the pedicle of Rhynchonelliformea [85, 90]. Further investigation of the postmetamorphic expression of *Hox* genes, especially *lox4* and *antp*, in *N. anomala* could support this hypothesis.

Unlike some bilaterians in which metamorphosis seems to be related to highly different *Hox* gene expression between larvae and adults (e.g., tunicates [15], Bryozoa [91], scaphopods [52]) or in which *Hox* genes are not expressed in the larvae and only pattern adult body (pilidiophoran nemerteans [49], indirectly developing Hemichordates [50], sea urchins [51, 53, 54]), rhynchonelliformean brachiopods exhibit continuity in the patterning of larval and adult body plans. Consequently, in regard to *Hox* gene expression, metamorphosis in *T. transversa* is similar to the condition found in another spiralian clade, Annelida. Although there are some shifts in expression patterns of particular *Hox* genes between annelid larvae and juvenile worms [47, 48], those differences are mostly related to restriction of some of the genes from broader larval to more specific adult domains [47]. This similarity can be explained if one assumes that, same as in Annelida, the metamorphosis of rhynchonelliformean larvae is not as drastic as it might seem and instead represents a relatively gradual process [67]. In *T. transversa*, several of the adult structures, including shell secreting epithelium [64, 68] or pedicle muscles [70, 72], are already present in the competent larvae as the Anlagen. Thus, even though transition from larva to juvenile poses large ecological change, from the morphological point of view the mantle lobe inversion is related mostly to tissue relocation and not to the degeneration or formation of entire body regions, as is the case in pilidiophoran nemerteans, indirectly developing hemichordates and sea urchins or ascidians.

From the phylogenetic and developmental point of view, it would be interesting to compare shifts of *Hox* genes expression observed during metamorphosis between *T. transversa* and Phoronida. Phoronids are closely related to brachiopods [57, 58, 92, 93] (in past even proposed as specialized clade belonging to Brachiopoda [62, 82]) and their rapid metamorphosis involves drastic rearrangements of the larval body plan [67, 94–97], which is much more complicated than the transition found in Rhynchonelliformea and sometimes referred to as catastrophic or cataclysmic metamorphosis [94, 96, 98]. The recent analysis of the body region-specific transcriptomes revealed that in adults of *Phoronis austarlis*, which possesses an organized *Hox* cluster, *Hox* gene expression does not exhibit spatial collinearity [18]. Unfortunately, data on the spatial expression of *Hox* genes in early developmental stages of any phoronid species are still lacking [98], preventing analysis of metamorphosis-related *Hox* genes expression shifts. Nevertheless, it is possible that in phoronids the larvae and juveniles exhibit pronounced differences in the *Hox* genes expression as is a case in some other animals with catastrophic and extensive metamorphosis [15, 49, 50].

### Germ layer-specific expression of *Hox* genes

In most of the investigated Bilateria, *Hox* genes are predominantly expressed in the ectodermal domains and often their antero-posterior staggered expression is especially evident in the neuroectoderm, which lead to the assumption that at least one of the original roles of *Hox* genes was patterning of the developing nervous system along A-P axis [27, 32, 99]. Interestingly, we did not find any of the *Hox* genes expressed in the nervous system of postmetamorphic juveniles of *T. transversa*. This could be explained by the fact that in juveniles the main nervous structures are brain and lophophore nerves (Fig. 2C) both related to the larval anterior lobe and postmetamorphic lophophore rudiment, which represent derivatives of the head and hence do not express *Hox* genes (the same has been shown for the phoronid lophophor [18]). Schiemann et al. [19] also did not describe expression of any of the Hox genes in neuroectoderm of earlier developmental stages of *T. transversa*. However, as co-expression of neuroectoderm markers has not been tested in that work, it is difficult to ascertain whether *T. transversa* really lack *Hox* genes expression in neuroectoderm on all developmental stages.

*Hox* genes can be also expressed in particular mesodermal domains in almost all investigated bilaterians, with the exception of Hemichordates (where their expression is restricted to ecto- and endoderm [29, 50]), rotifers (expression exclusively in the nervous system [32]) and Nemerteans (expression in ecto- and neuroectoderm [41]). Whether *Hox* genes were ancestrally expressed in the bilaterian mesoderm remains an open question. Nevertheless, taking into account that set of *Hox* genes expressed in the mesodermal derivatives differs substantially from one animal group to another and that their transcription in mesodermal tissues can happen on very different developmental stages, it seems plausible that *Hox* genes have been recruited many times independently to act in mesoderm development and specification [99].

In Brachiopods, three of the *Hox* genes (*pb*, *hox3* and *dfd*) show mesodermal expression albeit all of them are also expressed in ectodermal domains at some point of development [19]. Prior to the metamorphosis, *hox3* and *dfd* are expressed both in the mesodermal and ectodermal structures and after metamorphosis, due to the restriction of broader domains into specific structures, *hox3* remained expressed only in the ectoderm, whereas *dfd* become restricted to the mesoderm (Fig. 7).

Orthologs of those three genes are reported as mesodermally expressed in some other spiralian species as well. For instance, *pb* exhibits mesodermal expression domains in gastropod *Haliotis asinina* [36] and two annelids—*Chaetopterus variopedatus* [33] and *Alitta virens* [48], *hox3* and *dfd* are expressed in the mesoderm of scaphopod *Antalis entalis* [52] and *hox3* is mesodermally expressed in annelid *Capitella teleta* [47]. However, lack of evidence that all three of those three genes are expressed in the mesoderm of any single non-brachiopod spiralian species as well as different timing of their mesodermal expression in particular animals indicates that expression of *pb*, *hox3* and *dfd* in developing mesoderm might represent apomorphic feature of Brachiopoda or Lophophorata (investigation of phoronids and ectoprocts is needed to ascertain).

### Expression of *Hox* genes during the morphogenesis of brachiopod-specific structures in *T. transversa*

Although *Hox* genes are believed to originally be responsible for antero-posterior patterning [1, 2, 11], in certain animal lineages some of them were co-opted for morphogenesis of evolutionary novel structures [100–103]. Among Spiralia, such phenomenon has been reported in, e.g., conchiferan molluscs [36–38, 52] and annelids [34, 35, 47, 48], whereas recently Schiemann et al. suggested that in brachiopod larvae 4 out of 10 *Hox* genes have been recruited for patterning of chaetae (*lab* and *post1*) and shell fields (*scr* and *antp*) [19]. Our results generally support findings of Schiemann et al.—although we did not find evidence for the expression of *antp* in the shell field—and show that co-option of *Hox* genes for morphogenesis of novel structures is even more pronounced in juveniles of *T. transversa* than it is in the larvae.

*lab* and *post1* are recruited for the morphogenesis of chaetae in the embryos and larvae of *T. transversa* [19]. The gene *lab* is constantly expressed in the chaetal sacs from formation of their early Anlagen up to the latest larval stage, whereas *post1* is only briefly expressed during short time window, when the Anlagen are formed. In our study, we detect expression of *lab* in the chaetal sacs of juveniles as well, but surprisingly we found that *post1* is also expressed in the postmetamorphic juveniles. Moreover, its expression is not only restricted to the chaetal sacs but instead could be detected in the entire marginal zone of the mantle. This finding, however, makes sense when one takes into consideration that as adults *T. transversa*, as most of the rhynchonelliformean brachiopods, possess numerous chaetae along the mantle margin [88, 104]. We therefore propose that although both *lab* and *post1* are involved in the chaetae formation in Rhynchonelliformea, they play different roles: *post1* is expressed in the regions where prospective chaetae will develop, possibly stimulating epidermal cells to differentiate into chaetal sacs before its expression decays. A similar role has been suggested for *post1* in annelids, whose chaetae are considered homologous to brachiopod ones based on morphological [105] and molecular [19] similarities. In annelids, *post1* is expressed in the cells of developing chaetae-bearing parapodia, but the expression becomes more faint over the time of development and is not detectable in the already formed parapodia [34, 35, 47]. *lab*, on the other hand, is possibly involved in the patterning of the growth of the chaetae itself, remaining expressed long after onset of chaetal sac formation. This hypothesis needs to be tested in the future by functional gene inference and the examination of older juveniles or adults, in which, accordingly, we would expect lack of *post1* expression and broad expression of *lab* along the entire mantle margin.

Additionally, our investigation of the expression of chaetae-related genes in the earlier developmental stages of *T. transversa* shows that process of chaetal sacs formation is complicated and involves cell types, which spatially and temporarily differ in their gene expression profiles. At the late gastrula stage, *lab*, *post1* and *arx* are all expressed in the two pairs of cell clusters, which have been interpreted as chaetal sacs Anlagen by Schiemann et al. [19]. In the later larval stage, only expression of *lab* is retained in the chaetal sacs-related cells, *post1* is not expressed anymore, whereas expression of *arx* is shifted to the inner mantle lobe epithelium, which secrets protegulum (the larval shell rudiment). Interestingly, *arx* is not only expressed in the chaetal sacs Anlagen of annelids [81] and brachiopods and in the protegulum secreting epithelium of brachiopods but also in the radula formative tissue of the gastropod *Tylomelania sarasinorum* [106]. This indicates that among lophotrochozoans *arx* is generally expressed in the tissues forming various hard structures and cannot be unambiguously related to only single type of them.

The two-valved shell and posterior pedicle represent two distinct apomorphies of brachiopods, and we found four out of ten *Hox* genes expressed in the structures related to those morphological novelties. Our results indicate that *scr* is likely co-opted for the juvenile shell formation, as the gene is expressed in the mantle margin in the region specialized for shell secretion [69]. This finding corresponds to the results of Schiemann et al. [19], who found expression of *scr* in the epithelial cells forming larval shell rudiment.

Both shell and pedicle require sets of specialized muscles, which constitute an important part of the brachiopod body. In the late larvae of *T. transversa*, the genes *pb* and *dfd* are likely responsible for A-P patterning of mesoderm [19], yet during postembryonic development they seem to be recruited into morphogenesis of specific muscular structures that drive the biomechanics of, respectively, shell and pedicle. Additionally, *hox3*, another gene that seems to play a role in mesoderm patterning during earlier developmental stages [19], is expressed in the regions where future hinge rudiments will develop [69], suggesting that it could be involved in the morphogenesis of this autapomorphic rhynchonelliformean feature.

## Conclusions

All developmental stages of *T. transversa*, including juveniles, express *Hox* genes in a spatially non-collinear manner [19]. Most of the patterns observed in the late larvae seem to persist throughout metamorphosis and are retained in juveniles, corroborating morphological observations that metamorphosis, despite being rapid, is of gradual type and most of the adult organs are present as Anlagen in the competent larvae. The most drastic shifts in *Hox* gene expression patterns observed during metamorphosis can be explained by: (1) the inversion of the mantle lobe which relocates some of the more posterior larval structures into the anterior edge of the juveniles and (2) restriction of the broad expression domains, present in larvae, to the specific structures in juveniles.

Concordantly to the previous study on larvae of *T. transversa*, we found that certain *Hox* genes have been evolutionary co-opted for morphogenesis of specialized structures in brachiopods. In both larvae and juveniles, *lab* is expressed in the chaetal sacs, whereas *post1* marks the area where prospective chaetae will develop. In juveniles, four out of the ten *Hox* genes are expressed in the epidermal (*scr*, *hox3*) and muscular (*pb*, *dfd*) tissues related to shell and pedicle, two autapomorphic features of Brachiopoda.

## Methods

### Animal collection and fixation

Gravid adults of *T. transversa* (Sowerby 1846) were collected near San Juan Island, Washington, USA. Eggs obtained from the animals were fertilized, and developing larvae were cultured following previously published protocols (e.g., [19, 64, 68]) up to the metamorphosis. Two days after metamorphosis, juvenile animals were gently scraped from the bottom of the dish with a razor blade, relaxed with MgCl_2_, fixed in 3.7% formaldehyde and washed in phosphate buffer. Fixed animals were stored in 100% methanol.

### In situ hybridization

Probes against *Hox* genes were synthesized using the same plasmid clones as used in Schiemann et al. [19], where the gene orthology assessment has been performed. Single whole-mount in situ hybridization was performed following an established protocol [107]. dUTP-digoxigenin-labeled probes were hybridized at a concentration of 1 ng/μl at 67 °C for 72 h, detected with anti-digoxigenin-AP antibody in 1:5000 concentration in blocking buffer and visualized with nitroblue tetrazolium chloride and 5-bromo-4-chloro-3-indolyl phosphate (in colorimetric in situ hybridization) or detected with anti-digoxigenin-POD antibody in 1:200 concentration in blocking buffer and visualized with TSA-Cy5-Plus (in fluorescent in situ hybridization). Additionally, animals prepared for FISH were stained for 30 min in DAPI to visualize cell nuclei. Stained juveniles where mounted in 70% glycerol and examined with Zeiss Axiocam HRc connected to a Zeiss Axioscope Ax10 using bright-field Nomarski optics (CISH) or scanned in Leica SP5 confocal laser scanning microscope (FISH). Double fluorescence in situ hybridization was conducted as described elsewhere [108].

### Immunohistochemistry

For investigation of juvenile morphology, mouse primary monoclonal antibodies against tyrosinated-tubulin (Sigma, T9028) and acetylated-tubulin (Sigma, T6793) were used in 1:500 concentration. To visualize the primary antibodies, secondary goat anti-mouse antibodies (Life Technologies) conjugated with fluorochrome (AlexaFluor647) were applied in 1:50 concentration. F-actin was visualized with AlexaFluor555-labeled phalloidin, and cell nuclei were stained with DAPI. Stained juveniles were mounted in 80% glycerol and scanned in Leica SP5 confocal laser scanning microscope.

### Image processing and figure preparation

*Z*-stacks of confocal scans were projected into 2D images and 3D reconstructions in IMARIS 9.1.2. Both light micrographs and CLSM images were adjusted in Adobe Photoshop CS6 and assembled in Adobe Illustrator CS6. All the schematic drawings were done with Adobe Illustrator CS6.

## Additional file

**Additional file 1: Fig. S1.** Background signal resulting from unspecific binding of probes by surface of larval dorsal protegulum (asterisks) and borders between protegulum and remaining shell (arrowheads). The control without probes (A). Colorimetric in situ hybridization with sense probe of *antp* (B) and *post2* (C) genes, signal developed for the same time as for antisense probes. Fluorescent in situ hybridization with antisense probes of *antp* (D, E) and *post2* (F, G) genes, on E and G combined with DAPI staining of cell nuclei. Dorso-ventral view with anterior to the top (A–D, F) and virtual cross section with dorsal to the top (E, G). Dashed lines with letters on D and F indicate section planes shown on respective plates
