# Supplementary figures and images for: Hox gene expression in postmetamorphic juveniles of the brachiopod Terebratalia transversa
Source: EvoDevo. 2019 Jan 8;10:1. doi: 10.1186/s13227-018-0114-1 (PMC6325747; doi:10.1186/s13227-018-0114-1)

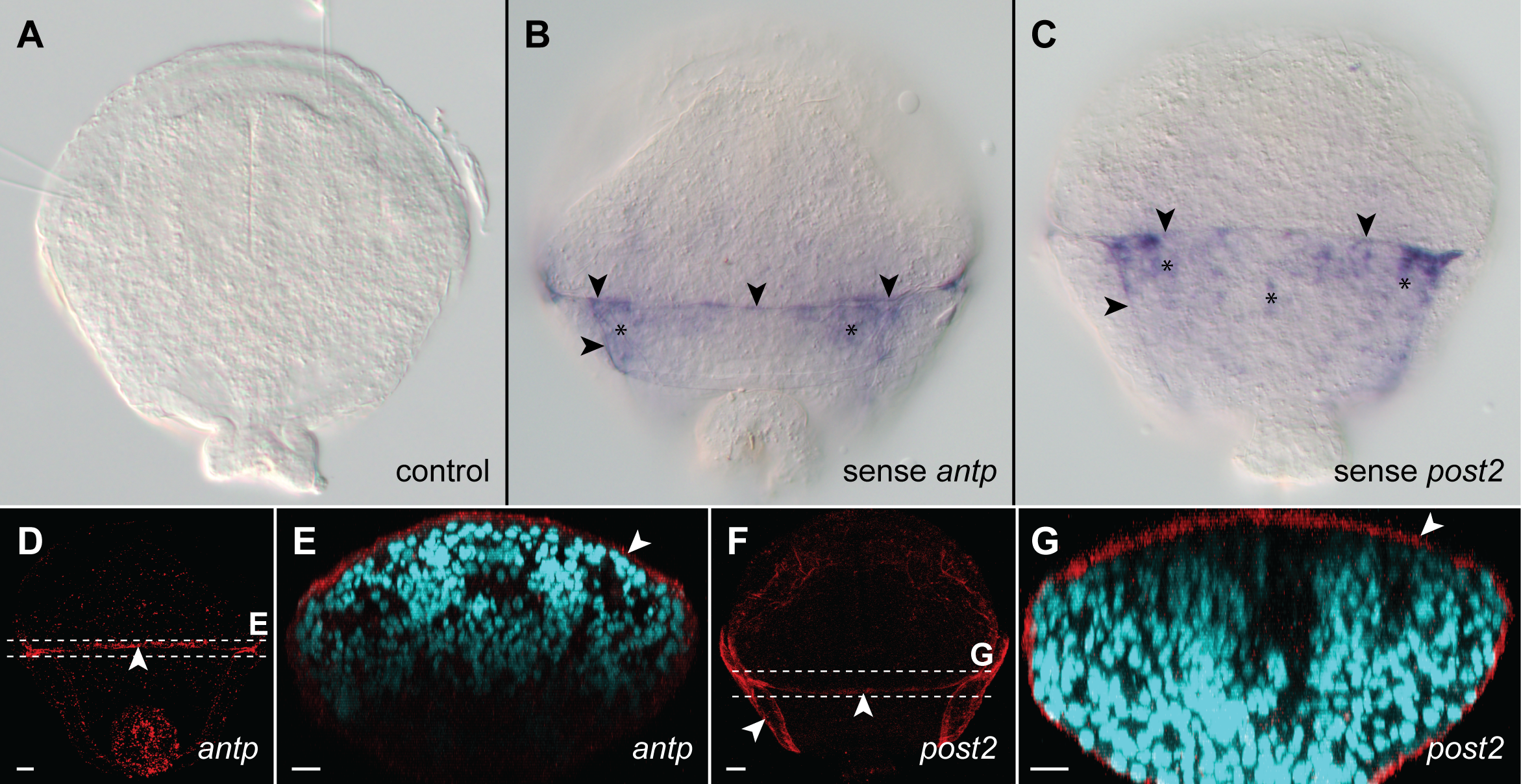

Supplement: Supplementary file 1 — Additional file 1: Fig. S1. Background signal resulting from unspecific binding of probes by surface of larval dorsal protegulum (asterisks) and borders between protegulum and remaining shell (arrowheads). The control without probes (A). Colorimetric in situ hybridization with sense probe of antp (B) and post2 (C) genes, signal developed for the same time as for antisense probes. Fluorescent in situ hybridization with antisense probes of antp (D, E) and post2 (F, G) genes, on E and G combined with DAPI staining of cell nuclei. Dorso-ventral view with anterior to the top (A–D, F) and virtual cross section with dorsal to the top (E, G). Dashed lines with letters on D and F indicate section planes shown on respective plates [file 13227_2018_114_MOESM1_ESM.tif]
